# Sequence Analysis of 96 Genomic Regions Identifies Distinct Evolutionary Lineages within CC156, the Largest *Streptococcus pneumoniae* Clonal Complex in the MLST Database

**DOI:** 10.1371/journal.pone.0061003

**Published:** 2013-04-12

**Authors:** Monica Moschioni, Morena Lo Sapio, Giovanni Crisafulli, Giulia Torricelli, Silvia Guidotti, Alessandro Muzzi, Michèle A. Barocchi, Claudio Donati

**Affiliations:** Research Center, Novartis Vaccines and Diagnostics, Siena, Italy; Centers for Disease Control & Prevention, United States of America

## Abstract

Multi-Locus Sequence Typing (MLST) of *Streptococcus pneumoniae* is based on the sequence of seven housekeeping gene fragments. The analysis of MLST allelic profiles by eBURST allows the grouping of genetically related strains into Clonal Complexes (CCs) including those genotypes with a common descent from a predicted ancestor. However, the increasing use of MLST to characterize *S. pneumoniae* strains has led to the identification of a large number of new Sequence Types (STs) causing the merger of formerly distinct lineages into larger CCs. An example of this is the CC156, displaying a high level of complexity and including strains with allelic profiles differing in all seven of the MLST loci, capsular type and the presence of the Pilus Islet-1 (PI-1). Detailed analysis of the CC156 indicates that the identification of new STs, such as ST4945, induced the merging of formerly distinct clonal complexes. In order to discriminate the strain diversity within CC156, a recently developed typing schema, 96-MLST, was used to analyse 66 strains representative of 41 different STs. Analysis of allelic profiles by hierarchical clustering and a minimum spanning tree identified ten genetically distinct evolutionary lineages. Similar results were obtained by phylogenetic analysis on the concatenated sequences with different methods. The identified lineages are homogenous in capsular type and PI-1 presence. ST4945 strains were unequivocally assigned to one of the lineages. In conclusion, the identification of new STs through an exhaustive analysis of pneumococcal strains from various laboratories has highlighted that potentially unrelated subgroups can be grouped into a single CC by eBURST. The analysis of additional loci, such as those included in the 96-MLST schema, will be necessary to accurately discriminate the clonal evolution of the pneumococcal population.

## Introduction

Disease-causing pneumococci represent a phenotypically and genotypically diverse population of strains that cause bacteraemia, meningitis, pneumonia, sinusitis, and acute otitis media in children [1]–[4]. Effective epidemiological surveillance, along with the characterization and the classification of the circulating strains, are important tools to understand the evolution and the population dynamics that support the success of specific pneumococcal lineages [5]–[9]. The capsule, of which there are 94 known types, is the most important pneumococcal virulence factor, as well as the target of current licensed vaccines and the most widely used single parameter for epidemiological typing of pneumococcal strains [10]–[14].

Within recent years, the accessibility of sequence analysis tools has increased the diffusion of *Streptococcus pneumoniae* molecular genotyping methods such as Multi Locus Sequence Typing (MLST) (http://spneumoniae.mlst.net/) [15], [16]. *S. pneumoniae* MLST is based on sequence data from standardized fragments of seven housekeeping genes; each unique allele is identified by a numerical ID, and the allelic profile at the seven loci is used to classify bacterial isolates into Sequence Types (STs) [17]. The MLST classification reveals important insights into the geographic spread of successful pathogenic clones and also the emergence of associations between STs and serotypes, combinations that can be traced back to serotype switching events [18]–[22].

The use of specifically designed algorithms such as eBURST, shows that MLST-related strains can be further grouped into Clonal Complexes (CCs) ideally including only genotypes descending from a common predicted founder [23], [24]. This type of analysis allows studying the temporal evolution of lineages with respect to capsular serotype switch events, antibiotic resistance acquisition and geographic distribution [5], [18], [25]. In addition, isolates belonging to the same ST or CC have been demonstrated to inherit specific genetic traits, like the two pilus encoding islets (*PI-1* and *PI-2*), the pneumococcal pathogenicity island *psrP-secY2A2,* and *pcpA* (Pneumococcal choline binding protein A) [26]–[31]. Recently, formerly distinct CCs have merged into larger and extremely heterogeneous CCs due to the extensive analysis of *S. pneumoniae* strain collections from different geographical regions and the consequent identification of several rare STs. One example is CC156 (the largest clonal complex in the MLST database), whose predicted founder based on eBURST analysis is ST156, although a recent report based on its penicillin susceptibility profile suggests that ST162 is the true ancestral ST [25].

Whether or not CC156 still represents the evolutionary descent from a predicted ancestor or is an example of artificial grouping due to the reduction of the discriminatory power of the eBURST algorithm, is a matter of scientific interest. The application of alternative typing methods with respect to MLST would be of paramount importance to unravel this point. Whole genome sequence analysis has been successfully applied to *S. pneumoniae* and has provided a clear indication that pneumococcal genomic variability is most likely triggered by homologous recombination events [32], [33]. In addition to whole genome sequencing, other molecular typing methods such as multiple-locus variable number tandem repeat analysis (MLVA), multilocus boxB sequence typing (MLBT) and 96-MLST, able to trace capsular switch or other homologous recombination events, have been proposed [15], [34]–[36].

In order to assess whether distinct genetic lineages were present in CC156, we applied 96-MLST to a panel of strains representative of 41 different STs belonging to CC156. The application of the 96-MLST schema allowed for the distinction of ten lineages within CC156, each homogeneous in terms of capsular type and for the presence of clonally inherited genetic traits (such as the presence of PI-1). Noteworthy, strains belonging to ST4945, whose recent discovery had been responsible for the merging of distinct clones into CC156, were unequivocally assigned to one of the lineages. Moreover, two out of the three ST4945 SLV analyzed were assigned to lineages different from ST4945, suggesting that strains in close proximity in the eBURST graphic representation (and differing in only one of the seven MLST alleles) can be significantly different when considering additional loci interspersed in the whole genomic backbone.

## Materials and Methods

### Strain Collection

The collection of *S. pneumoniae* clinical isolates analysed in this study was representative of the allelic (ST) diversity of CC156 strains. The following information on the 66 strains used in this study is listed in Table 1: name, serotype, ST, geographical origin, the number of MLST alleles in common with ST156 (the CC156 eBURST predicted founder) and with ST4945, data source and strain source. Overall, the strains analyzed belong to 41 CC156 STs and are associated with 14 capsular types. The isolate collection was composed of 8 strains for which either complete or draft genomic sequence was available and 58 additional isolates for which new sequence data were generated (*see* below). The 58 isolates already characterized for serotype and ST were kindly provided by many laboratories (complete list in the “Acknowledgments” section).

**Table 1:** CC156 strain panel used in this study.

Strain name	ST	Serotype/serogroup	Country	MLST alleles in common with ST4945	MLST alleles in common with ST156	PI-1	Data source	Strain source	Lineage
**6B 670**	90	6B	Spain	2/7	1/7	yes	GenBank:CP002176	http://jb.asm.org/content/189/22/8186.long	f
**PT134**	94	6B	Italy	2/7	1/7	yes	This Study	Istituto Superiore di Sanità, Italy	f
**CCRI 1974**	124	14	Canada	4/7	1/7	no	GenBank:ABZC00000000	http://genome.cshlp.org/content/19/7/1214.long	d
**CCRI 1974M2**	124	14	Canada	4/7	1/7	no	GenBank:ABZT00000000	http://genome.cshlp.org/content/19/7/1214.long	d
**SP14**	124	14	USA	4/7	1/7	no	GenBank:ABAD00000000	http://jb.asm.org/content/189/22/8186.long	d
**2610-99**	138	6B	USA	3/7	1/7	yes	This Study	Center for Disease Control and Prevention, USA	b
**AP191**	143	14	Italy	3/7	5/7	yes	This Study	Istituto Superiore di Sanità, Italy	i
**6BIJ**	145	6B	Iceland	5/7	3/7	yes	This Study	Landspitali, National University Hospital of Iceland, Iceland	e
**1071**	146	6B	New Zeland	4/7	2/7	yes	This Study	Center for Disease Control and Prevention, USA	e
**1300023**	156	14	Israel	3/7	7/7	yes	This Study	Ben-Gurion University of the Negev, Israel	i
**1300025**	156	14	Israel	3/7	7/7	yes	This Study	Ben-Gurion University of the Negev, Israel	i
**1309530**	156	11A	Israel	3/7	7/7	yes	This Study	Ben-Gurion University of the Negev, Israel	i
**08B02945A**	156	9V	Thailand	3/7	7/7	yes	This Study	Shoklo Malaria Research Unit, Thailand	i
**08B02946A**	156	9V	Thailand	3/7	7/7	yes	This Study	Shoklo Malaria Research Unit, Thailand	i
**405A**	156	14	Brazil	3/7	7/7	yes	This Study	Oswaldo Cruz Foundation Salvador, Brazil	i
**635A**	156	14	Brazil	3/7	7/7	yes	This Study	Oswaldo Cruz Foundation Salvador, Brazil	i
**AP207**	156	9V	Italy	3/7	7/7	yes	This Study	Istituto Superiore di Sanità, Italy	i
**PT051**	156	14	Italy	3/7	7/7	yes	This Study	Istituto Superiore di Sanità, Italy	i
**PT052**	156	9V	Italy	3/7	7/7	yes	This Study	Istituto Superiore di Sanità, Italy	i
**PT094**	156	14	Italy	3/7	7/7	yes	This Study	Istituto Superiore di Sanità, Italy	i
**RP1554**	156	9V	Sweden	3/7	7/7	yes	This Study	Karolinska Institutet, Sweden	i
**RP3718**	156	9V	Sweden	3/7	7/7	yes	This Study	Karolinska Institutet, Sweden	i
**SP195**	156	9V	Worldwide	3/7	7/7	yes	GenBank:ABGE00000000	Genome Biol 11:R107	i
**274A**	162	9V	Brazil	4/7	6/7	yes	This Study	Oswaldo Cruz Foundation Salvador, Brazil	i
**35A**	162	9V	Brazil	4/7	6/7	yes	This Study	Oswaldo Cruz Foundation Salvador, Brazil	i
**PN131**	162	24F	Italy	4/7	6/7	yes	This Study	Istituto Superiore di Sanità, Italy	i
**PN314**	162	24F	Italy	4/7	6/7	yes	This Study	Istituto Superiore di Sanità, Italy	i
**1965-00**	166	9V	USA	4/7	6/7	Yes	This Study	Center for Disease Control and Prevention, USA	i
**BG02112**	171	6B	n.d.	3/7	2/7	no	This Study	University of Alabama, USA	b
**1300044**	172	23F	Israel	1/7	1/7	no	This Study	Ben-Gurion University of the Negev, Israel	a
**1309497**	172	19A	Israel	1/7	1/7	no	This Study	Ben-Gurion University of the Negev, Israel	a
**1309804**	172	23F	Israel	1/7	1/7	yes	This Study	Ben-Gurion University of the Negev, Israel	a
**08B09744**	172	23F	Thailand	1/7	1/7	no	This Study	Shoklo Malaria Research Unit, Thailand	a
**09B10384**	172	23F	Thailand	1/7	1/7	yes	This Study	Shoklo Malaria Research Unit, Thailand	a
**23F Poland-16**	173	23F	Poland	2/7	2/7	yes	This Study	Center for Disease Control and Prevention, USA	c
**PB011**	176	6B	Italy	2/7	1/7	yes	This Study	Istituto Superiore di Sanità, Italy	b
**2683-05**	239	9V	Poland	1/7	1/7	no	This Study	National Medicine Institute, Poland	g
**Hungary19A-6**	268	19A	Hungary	1/7	1/7	yes	GenBank:CP000936	Genome Biol 11:R107	c
**6BGreece-22**	273	6B	Greece	4/7	1/7	yes	This Study	Center for Disease Control and Prevention, USA	f
**08B08993**	280	9V	Thailand	2/7	1/7	no	This Study	Shoklo Malaria Research Unit, Thailand	g
**23F Colombia-26**	338	23F	Colombia	1/7	1/7	no	This Study	Center for Disease Control and Prevention, USA	a
**P1059**	361	6A	Ghana	2/7	2/7	no	This Study	Swiss Tropical Institute, Switzerland	a
**SPEC6B**	385	6B	USA	5/7	2/7	yes	This Study	University of Alabama, USA	e
**6037-01**	392	17F	USA	6/7	3/7	no	This Study	Center for Disease Control and Prevention, USA	h
**16-5**	440	23F	Italy	5/7	2/7	no	This Study	Ospedale le Scotte,Siena, Italy	h
**1404**	559	6B	Italy	2/7	1/7	Yes	This Study	Istituto Superiore di Sanità, Italy	b
**6541-97**	602	23F	Poland	4/7	1/7	no	This Study	National Medicine Institute, Poland	h
**4068-00**	642	9V	USA	4/7	4/7	Yes	This Study	Center for Disease Control and Prevention, USA	i
**6492-03**	671	14	USA	2/7	4/7	Yes	This Study	Center for Disease Control and Prevention, USA	i
**URUG2**	789	14	Uruguay	6/7	2/7	no	This Study	The Rockfeller University, New York, USA	d
**66994**	847	19A	Kenya	4/7	4/7	yes	This Study	Kenyan Medical Research Center, Kenya	j
**2805-02**	847	19A	Kenya	4/7	4/7	yes	This Study	Center for Disease Control and Prevention, USA	j
**SP9**	1269	9	USA	4/7	5/7	yes	GenBank:ABAB00000000	http://jb.asm.org/content/189/22/8186.long	i
**EU257**	1349	23B	Turkey	0/7	0/7	no	This Study	Center for Disease Control and Prevention, USA	a
**08B02447**	2218	23F	Thailand	2/7	1/7	no	This Study	Shoklo Malaria Research Unit, Thailand	a
**08B01504**	4404	6B	Thailand	6/7	3/7	no	This Study	Shoklo Malaria Research Unit, Thailand	e
**08B01829**	4405	6B	Thailand	5/7	2/7	no	This Study	Shoklo Malaria Research Unit, Thailand	e
**1464**	4945	17F	Sweden	7/7	3/7	no	This Study	Center for Disease Control and Prevention, USA	h
**1758**	4945	17F	Egypt	7/7	3/7	no	This Study	Center for Disease Control and Prevention, USA	h
**1789**	4948	14	Egypt	4/7	4/7	Yes	This Study	Center for Disease Control and Prevention, USA	i
**1623**	4966	6B	Thailand	4/7	3/7	No	This Study	Center for Disease Control and Prevention, USA	b
**1681**	4966	6C	Thailand	4/7	3/7	No	This Study	Center for Disease Control and Prevention, USA	b
**582**	4968	23A	Mozambique	1/7	1/7	No	This Study	Center for Disease Control and Prevention, USA	a
**770**	5420	6B	Thailand	4/7	3/7	No	This Study	Center for Disease Control and Prevention, USA	b
**1953**	5613	6A	Nepal	4/7	2/7	No	This Study	Center for Disease Control and Prevention, USA	b
**SP18**	6214	6	USA	3/7	2/7	yes	GenBank:ABAE00000000	http://jb.asm.org/content/189/22/8186.long	e

For each strain name, ST, serotype/serogroup, country of isolation, number of MLST alleles in common with ST156 and ST4945, data source, strain source and lineage (as identified by 96-MLST hierarchical clustering, *see* Figure 2) are indicated.

### Genomic DNA Extraction and PI-1 Presence Assessment

Bacteria were grown overnight at 37°C in 5% CO_2_ on Tryptic Soy Agar plates (TSA) (Becton Dickinson) supplemented with 10 mg/l colistine, 5 mg/l oxolinic acid and 5% defibrinated sheep blood. Genomic DNA extractions for the 58 isolates were performed by using the Wizard Genomic DNA purification kit following the manufacturer’s instructions (Promega). PCR amplifications for 96-MLST were performed on genomic DNA as briefly described below, while the presence of PI-1 and the PI-1 clade were determined as reported elsewhere [27].

### 96-MLST

The 96-MLST loci and the amplification primers for the 96-MLST schema reported in Tables S1 and S2 were selected and designed as detailed elsewhere [34]. Briefly, the 96 loci were identified within the *S. pneumoniae* core genes by comparing the available complete and draft genomes of 39 *S. pneumoniae* invasive strains and selected for being short variable regions (ranging from 847 to 209 bp) flanked by conserved regions. For the 8 complete and draft genomes (Table 1) of *S. pneumoniae* the nucleotide sequences of the 96 loci were retrieved by *in silico* analysis; a locus was considered to be absent when a primer aligned against the genome sequence with more than three mismatches to guarantee consistency with the results of the PCR amplifications. For this reason, the two loci SP0180 and SP0181 were not extracted from the SP195 genome.

The 96-MLST loci of the 58 *S. pneumoniae* isolates were PCR amplified in a microtiter 96-well plate by using a unique amplification condition (55°C annealing temperature, 1 minute elongation) with the primer set reported in Table S2 [34]. The PCR products were then purified with paramagnetic beads (Agentcourt AMPure XP, Beckman Coulter Genomics, USA) and both strands sequenced with the same amplification primers by an ABI 3730xl DNA Analyzer (Applied Biosystems, USA). Chromatogram traces were edited and assembled with Vector NTI Advance 11 (Life Technologies). Consensus sequences were aligned using MUSCLE 3.8.31 [37]. The SP0278, SP1785, SP1909, SP2141 and SP2198 loci were not amplified on 2, 4, 1, 1 and 3 strains respectively. The complete nucleotide sequence dataset is provided in the File S1.

### Hierarchical Clustering and Minimum Spanning Tree Analysis

Sequences were converted into allelic profiles (Table S3), by assigning a unique ID number to each allele. When a primer pair did not amplify, the absent locus was assigned the ID number “0″. With this choice, strains sharing the same deletions or divergent sequences in the primer region were considered more similar than what would be by treating these loci as missing data, thus allowing the use of the information in the phylogenetic analysis.

Hierarchical clustering was performed using the package Cluster v1.13.1 [38] of the software package R v2.12.0 (www.r-project.org/). Distances between strains were computed using the function “Daisy” with Gower’s distance, counting the number of differences between allelic profiles. An agglomerative hierarchical clustering of the data was performed using the function “Agnes” with “average” (unweighted pair-group average method – UPGMA) method. Support of the clustering was assessed using bootstrap.

Minimum Spanning Tree analysis was performed using PHYLOVIZ [39].

### Phylogenetic Analysis

For each of the 96 loci the sequences were aligned using MUSCLE [37]. Aligned sequenced where concatenated, and phylogenetic analysis was performed using Mega5 [40] with the Neighbor Joining method [41].

### ClonalFrame Analysis

ClonalFrame V1.1 [42] was run on the aligned sequences of the 89 loci that where present in all strains. Seven independent runs of 10^6^ iterations were performed, and the first half of each run was discarded. When compared, the distributions of some of the parameters from the seven runs failed to fulfill the convergence requirement of a Gelman and Rubin statistics below 1.2, suggesting that the evolutionary model implemented by ClonalFrame gives a poor description of these data. The posterior samples of the tree topologies of the seven runs were combined, and a consensus phylogenetic network was generated using SplitsTree v4.10.

## Results

### The Identification of ST4945 Strains has Been Sufficient to Cause the Merger of Three Independent *S. pneumoniae* Lineages into One Clonal Complex, CC156

**Figure 1:**
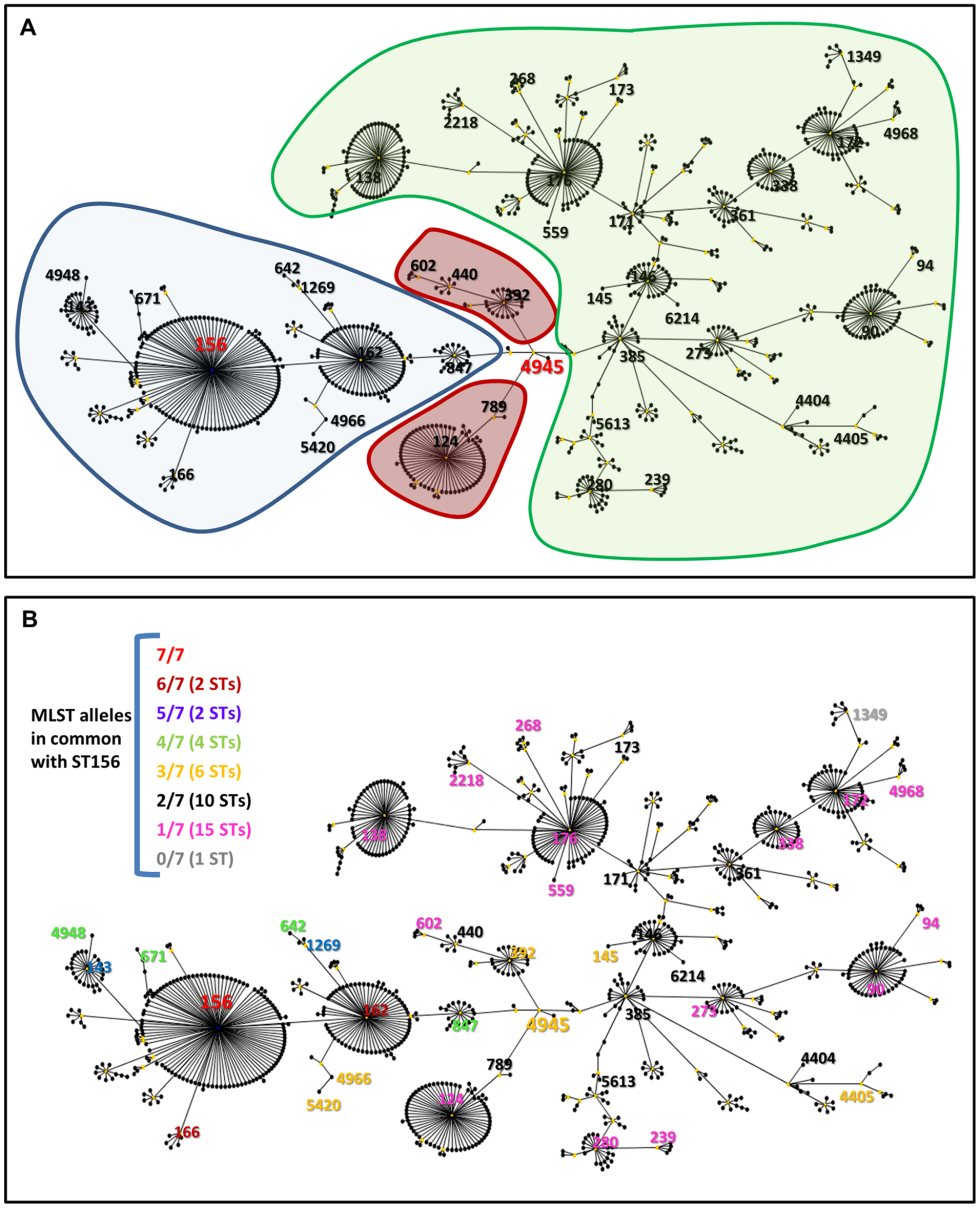
Graphic representation of CC156 by e-BURST. A) In the absence of ST4945 CC156 is partitioned in three different CCs by e-BURST analysis. B) 32 out of the 41 CC156 STs analyzed differ in four or more than four alleles from the founder ST, ST156. The MLST database was accessed on 15h January 2012 and CC156 visualized using eBURST (the e-BURST algorithm was executed on a dataset comprising all the STs in the database represented once). A) Shadowed shapes indicate the partitioning in distinct CCs of CC156 (CC162 blue, CC124 red, CC176 green) when eBURST was executed with the same ST dataset but excluding ST4945. ST156 and ST4945 are highlighted in red, while all the other STs analysed in this study are in black. B) The STs analysed in this study are highlighted and colour coded based on the number of MLST alleles in common with the predicted founder, ST156 (colour coding is indicated in the Figure).

CC156 (predicted founder ST156) was identified as the largest clonal complex in the *S. pneumoniae* MLST database (accessed on 15th January 2012) by running the eBURST algorithm with the default settings on the complete dataset which, at the time of the analysis, comprised 7146 distinct STs (ST1-ST7160). CC156 presented a complex and heterogeneous structure (Figure 1A and [25]), which did not change by using other algorithms such as goeBURST [23], encompassing 13.8% (986 STs) of the total STs in the database (as a comparison the second largest CC, CC320, included only 235 STs, 3.3% of the total). Interestingly, based on analyses performed in 2008, CC156 comprises STs which formerly belonged to distinct complexes: CC124, CC146, CC162, CC176, and CC392. These CCs were associated with different serotypes and differed for the presence of PI-1 and for PI-1 clade [27]. As detailed elsewhere [25], [27], [43], CC162 strains were associated with serotypes 9V and 14 and were PI-1 positive (PI-1 clade I), CC124 and CC392 strains were associated with serotypes 14 and 17F and were PI-1 negative, CC146 strains were associated with serotype 6B and PI-1 positive (clade II) and CC176 strains were generally associated with serotype 6B and 23F and heterogeneous for the PI-1 presence (clade II).

To investigate whether the merger of multiple CCs into a single CC was due to the identification of one or multiple new STs, the eBURST algorithm was iteratively executed by progressively excluding the most recently identified STs from the analysis. This resulted in the observation that ST4945 (a new allelic combination), occupied a central position within the eBURST CC156 graphic representation (Figure 1 and Figure S1), and had been sufficient to induce the merger of three formerly distinct CCs: CC162, CC124 and CC176 into one larger CC (Figure 1A). At the time of this analysis, only two ST4945, both serotype 17F from two different countries were present in the MLST database. Noteworthy, the CC124 and CC176 identified by excluding ST4945 from the analysis comprised the formerly separated complexes CC124 and CC392, and CC176 and CC146, respectively [27].

To further investigate whether the strains comprising the newly formed CC156 had a common evolutionary descendent (ST156) or whether ST4945 strains contained a combination of genetic alleles from different lineages (as suggested by their MLST profile), we analyzed a panel of 66 representative strains of different STs belonging to CC156 (see materials and methods section, Table 1, Figure 1 and Figure S1). The strains isolated in different countries belonged to 41 STs and shared between zero and six MLST alleles with ST156 (Figure 1B) despite belonging to the same CC. As shown in Figure S1A, all of the strains displayed the PI-1 distribution expected based on the lineage partitioning present before the introduction of ST4945 (*see* Figure 1A and [26], [27], [43]). The two ST4945 strains were also part of the collection as well as single and double locus variant (SLV, DLV) strains of ST4945 (Figure S1B).

### Hierarchical Clustering of the 96-MLST Alleles Identified Ten Genetically Distinct Evolutionary Lineages within the CC156 Strain Panel

**Figure 2:**
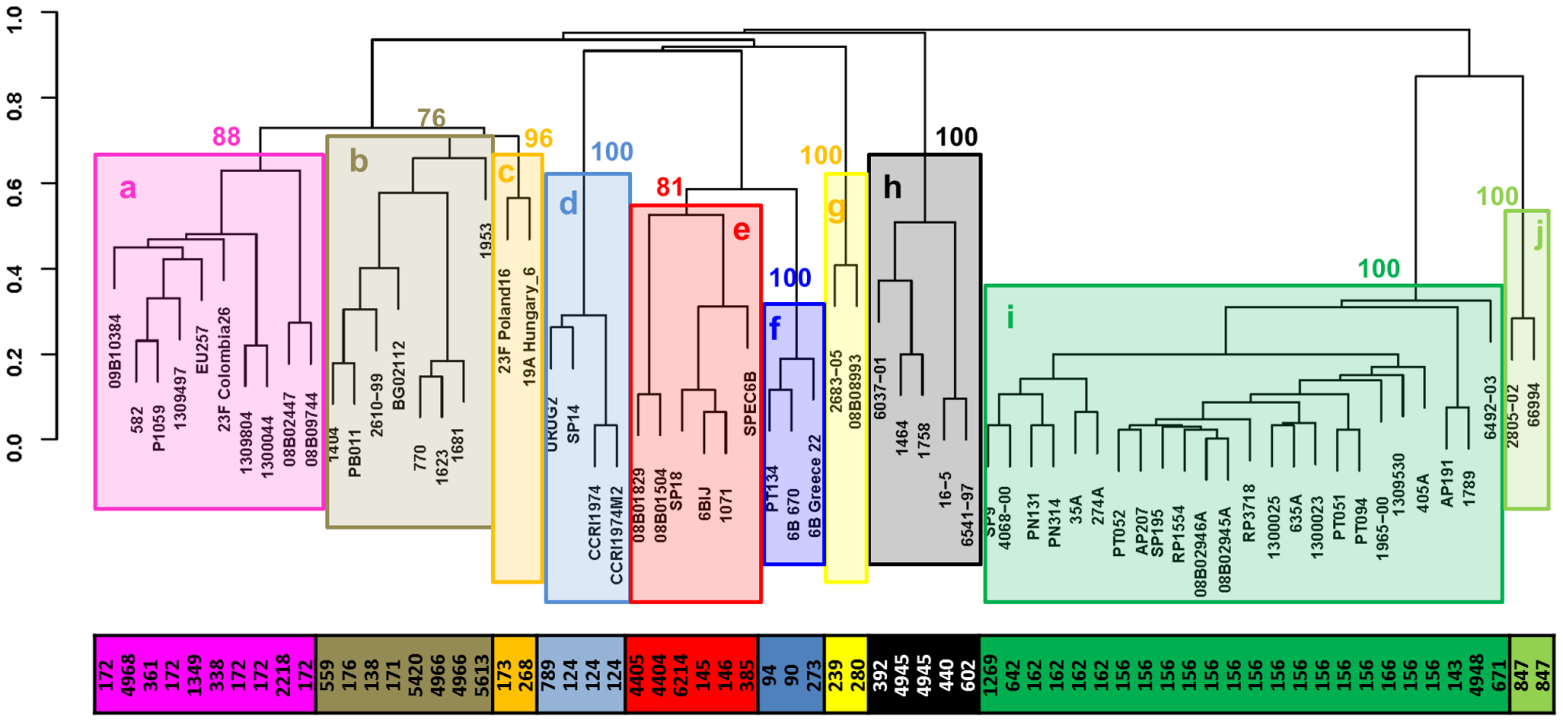
Hierarchical clustering performed on the 96-MLST alleles identifies ten genetically distinct evolutionary lineages (a-j) within the 66 CC156 strains analyzed. Sequences were converted into allelic profiles assigning a unique ID number to each allele. Hierarchical clustering was performed using the package Cluster v1.13.1. Distances between strains were computed using the function “Daisy” with Gower’s distance, counting the number of differences between allelic profiles. An agglomerative hierarchical clustering of the data was performed using the function “Agnes” with “average” (unweighted pair-group average method – UPGMA) method. The ten lineages identified (a-j) are indicated by coloured boxes, and numbers represent the bootstrap support. The STs of all the strains are indicated in the coloured bar.

The 66 strains were typed by using the 96-MLST schema [34]. Following amplification and sequencing, the sequences were converted into allelic profiles (Table S3). The minimum number of distinct alleles was identified in the SP0841 locus (6 alleles), while the maximum number of alleles was identified in the SP334 and SP2194 loci (28 alleles). By performing hierarchical clustering of the 66 strains using the number of loci with different alleles as a measure of their genetic distance it was possible to identify ten genetically distinct lineages supported by high values of bootstrap (Table 1, Figure 2 and Figure S2A). As shown in Figure 2, strains with the same ST were always assigned to the same lineage, while being SLV was not predictive for the assignment to the same lineage. Indeed, ST392, ST789 and ST4404 were all SLVs (in different MLST alleles) of ST4945 strains but only ST392 was assigned to the ST4945 lineage; the SLVs ST273 and ST385 were assigned to different lineages as well as the SLVs ST2218 and ST176; finally, ST361 and ST2218 (in a DLV relationship) both SLV of ST171 were allocated into the same lineage, different from that of ST171 (Figure 2). In addition, similar lineages could be identified also by using ClonalFrame (Figure S2B). Small differences (*e.g.* the position of strains 08809744 and 08802447) with respect to the results obtained with hierarchical clustering could be attributed to the fact that, despite having run 7 independent simulations for a total of 7*10^6^ iterations, incomplete sampling of the tree topologies was achieved. Remarkably, a phylogenetic analysis performed with the same dataset by aligning the 96 loci concatenated sequences, identified the same lineage pattern (Figure S3).

As expected, by computing a hierarchical clustering analysis with the MLST allelic profiles for the same set of 41 STs, SLV STs were grouped in the same lineage (Figure S4). This was also true when the phylogenetic analysis was performed with the sequences of the seven MLST loci (Figure S5). However, for the 7-MLST analysis the groupings obtained with the two methods were not exactly the same (i.e. for the two SLV STs 4966 and 5420).

Interestingly, the most remarkable differences in the hierarchical clustering between 96-MLST and 7-MLST were that lineages “d” and “h” (the latter comprising ST4945), “f” and “e”, “a” “b” and “c”, and “i” and “j” clustered together in the 7-MLST, thus justified by their STs proximity within the CC156 eBURST representation.

### ST4945 can be Assigned to One of the Identified Lineages by the 96-MLST Allelic Profile Analysis

**Figure 3:**
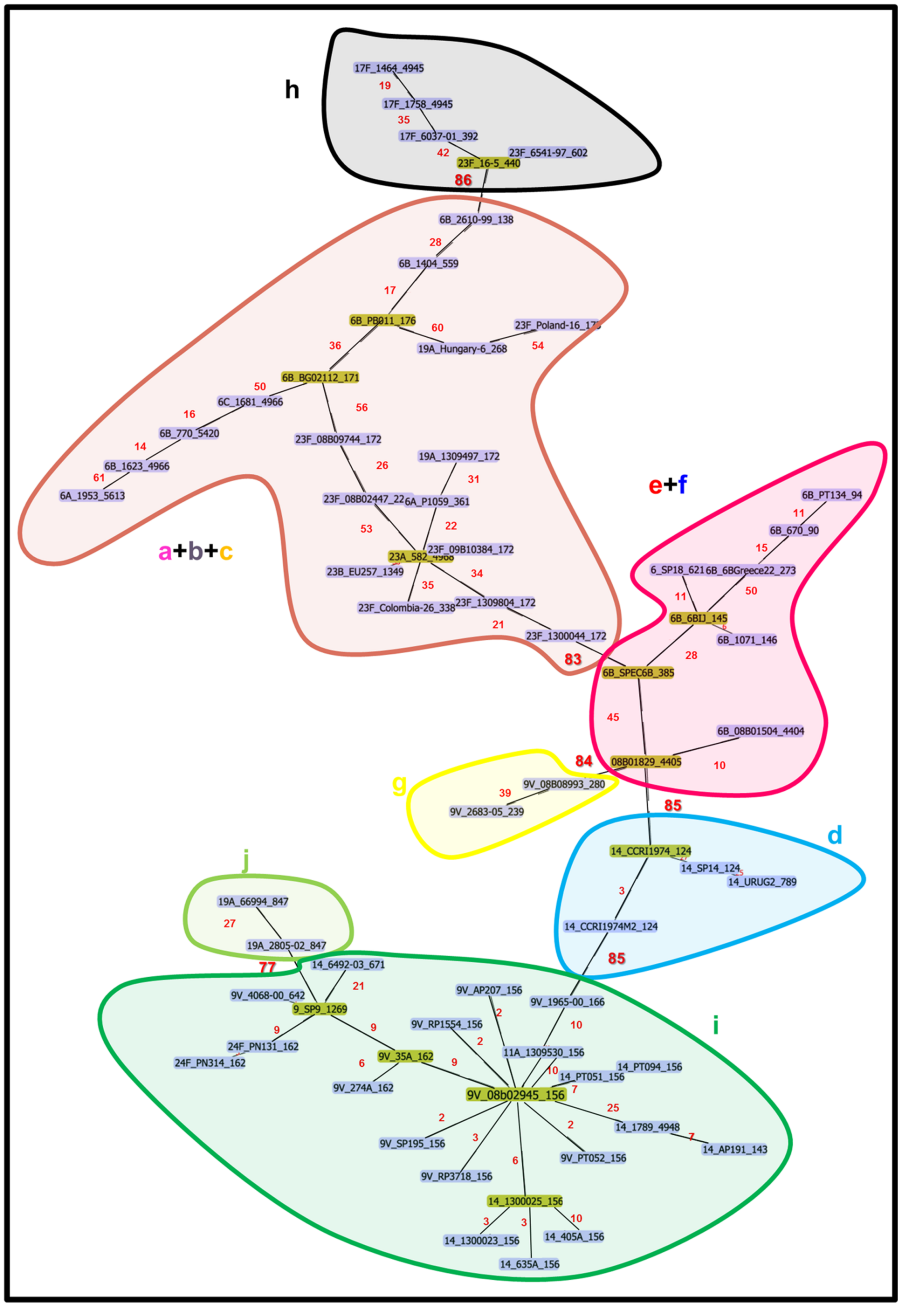
Minimum Spanning Tree analysis based on 96-MLST allelic profiles identifies seven distinct lineages by imposing a maximum threshold of 75 different loci. The Minimum Spanning Tree analysis was performed by using PHYLOVIZ on the 96-MLST alleles of the 66 strains considered in this study. The lineages identified by applying the threshold of 75/96 different loci are highlighted with shadowed shapes and named according to the lineage identification of Figure 2.

**Figure 4:**
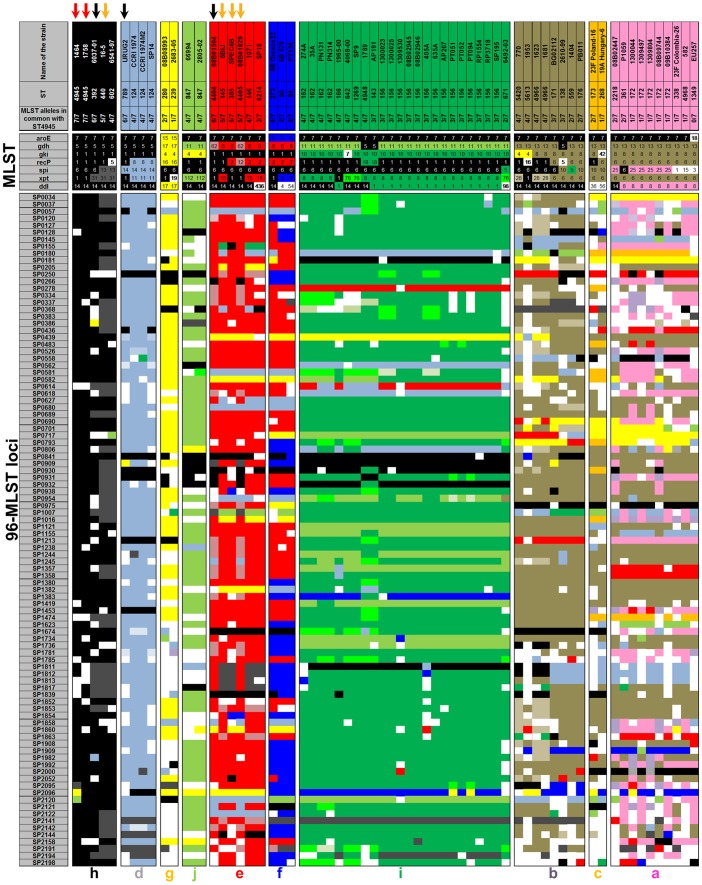
ST4945 can be unequivocally assigned to one of the identified lineages. The distribution of the 7-MLST and the 96-MLST alleles was analysed by assigning identical colours to identical alleles across the strains (white = unique alleles). Red arrows indicate ST4945 strains, while black and orange arrows indicate single and double 7-MLST locus variants of ST4945, respectively. The 96-MLST loci are listed according to their order in the genome.

The partitioning of CC156 into ten distinct lineages by hierarchical clustering was further evaluated by performing a Minimum Spanning Tree (MST) analysis of the 96-MLST alleles (Figure 3), and by creating a visual diagram of the allelic assortment within and across the identified lineages for both the 7 and the 96-MLST profiles (Figure 4). As shown in Figure 3, by applying a threshold of 75 loci (*i.e.* by cutting those links in the MST diagram that connected strains differing by more than 75/96 loci), seven distinct lineages could be identified. Notably, these lineages corresponded to those obtained with the hierarchical clustering analysis, although the strains belonging to lineages “a”, “b”, “c”, “e” and “f” were grouped by the MST analysis in two larger groups, “a+b+c” and “e+f”. Indeed, the lineages “a” and “b”, the lineages “b” and “c” and the lineages “e” and “f” were connected in the MST by strains differing in 56, 60 and 50 loci, respectively. In addition, the two ST4945 strains were unequivocally assigned to lineage “h” and the ST392 strain (SLV of ST4945 in the MLST classification) was the closest to the ST4945 strains according to 96-MLST, with 25 different alleles.

In order to visualize whether the allelic differences within and among lineages were concentrated in specific regions of the chromosome, and thus likely attributable to single recombination events, the strains were partitioned based on the lineages identified by hierarchical clustering analysis (Figure 4, columns). The alleles of the 7-MLST and 96-MLST loci (Figure 4, rows) were then colour coded by assigning the same colour to identical alleles within a locus. Since the rows in Figure 4 are ordered according to the position in the chromosome blocks of consecutive loci showing a colour pattern discordant with the rest of the chromosome or with other lineages are indicative of localized intra or inter-chromosomal differences, respectively. Overall, the distinction among genetic lineages was not due to differences present in specific hyper-variable regions of the chromosome. In fact, the differences were dispersed among the 96 loci and, apart from a few exceptions, each lineage contained a specific repertoire of alleles. Interestingly, lineages “d”, “e” and “h”, comprising ST4945 strains and SLV and DLV strains of ST4945 (black and blue arrows in Figure 4) shared several alleles in the loci probed by the 7-MLST typing schema, but were clearly distinct at the level of the 96-MLST loci. Besides, lineages “a”, “b” and “c” were the most heterogeneous, presented several unique loci and numerous alleles in common (thus justifying the fact they were classified into a unique cluster by MST and the lower support of the branching separating them in the hierarchical clustering and in the consensus network obtained from the posterior sampling of the tree topologies generated by ClonalFrame). A similar situation was shared by lineages “e” and “f”, although these two lineages presented a lower number of unique alleles.

**Figure 5:**
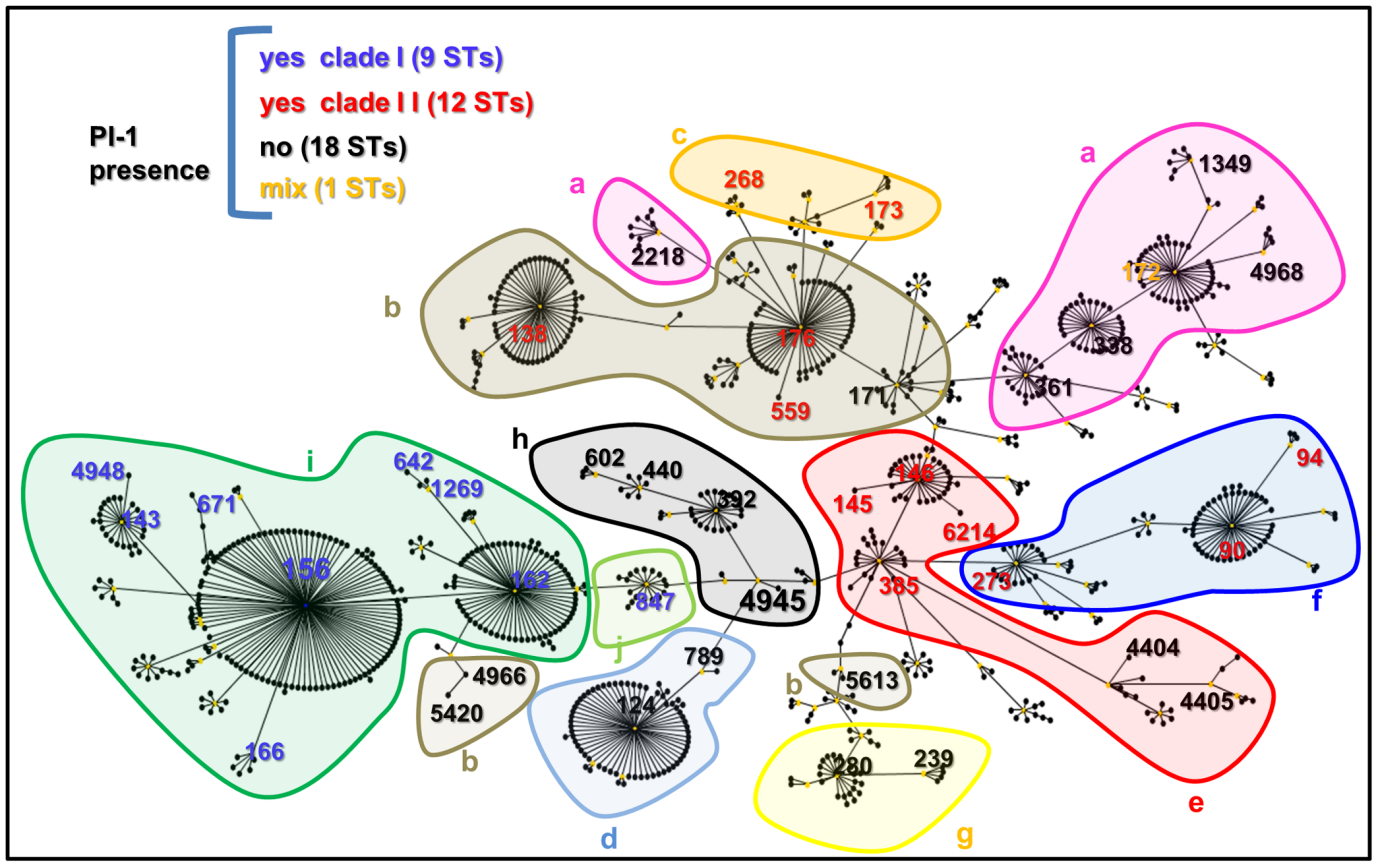
The CC156 lineages (a-j) identified with the hierarchical clustering (shadowed shapes) as defined in Figure 2 correlate with the ST distribution in the eBURST diagram and with PI-1 distribution. STs are indicated with different colours depending on PI-1 presence/absence as indicated in the Figure legend.

With the exception of lineages “a” and “b”, all of the strains belonging to the same lineage were also closely related in the eBURST graphic visualization of CC156 (Figure 5). In detail, ST2218, spatially separated from the other lineage “a” strains, is both SLV of lineage “a” ST172 and of lineage “b” ST176, but had 70 and 31 96-MLST loci in common with the closest ST172 strain 08b09744 (lineage “a”) and ST176 strain PB011 (lineage “b”), respectively. Besides, ST5613, ST4966 and ST5420 were separated from the other lineage “b” strains. Noteworthy, based on MST analysis, strains belonging to these three STs were closer to one another than to other strains in the collection, being the ST171 strain BG02112 the closest to the ST4966 strain 1681 (46/96 alleles in common).

In addition, as shown in Figure 5, the majority of the lineages were homogeneous for the presence/absence of PI-1 and, when PI-1 was present, strains within the same lineage carried, (as a confirmation of their phylogenetic proximity) the same PI-1 clade.

## Discussion

The implementation of increased regional surveillance combined with molecular typing methods has allowed for the identification of several successful *S. pneumoniae* clones with a higher invasive disease potential and an ability to spread across different geographical regions. These clones are variably associated with antibiotic resistance and some have been reported to rapidly evolve over time [5], [9], [16], [18], [44].

The genetic characterization of *S. pneumoniae* strains has contributed to the recent progress in pneumococcal biology; however, the genetic traits that allow for the success of specific clones and those responsible for the diversity observed in the *S. pneumoniae* population are still not completely identified. Out of the several typing methods available to characterize the pneumococcus, MLST is the most widely used for its ability to discriminate bacterial strains. In the MLST framework, relatedness among STs is inferred by methods of cluster reconstruction, or by simple models of clonal expansion and diversification to infer patterns of evolutionary descent, as done by eBURST. However, analyses of this nature must be undertaken with caution due to the potential for recombination events to obscure the evolutionary history of linked groups of strains. By reshuffling the MLST loci, recombination can produce combinations of alleles that cause the merger of unrelated lineages of clonal descent into large, heterogeneous CCs. The probability of the occurrence of these events increases when bacterial collections expand and large numbers of new STs are identified leading to a reduction of the discriminatory power and practical utility of the eBURST algorithm.

An example of this event is clonal complex CC156, which includes a large and heterogeneous group of strains that in many cases differ in all MLST loci, but nevertheless are connected by a continuous path of SLVs. In this report we provide evidence that the identification of a new ST (ST4945) was sufficient to induce the merger of formerly distinct CCs (here at least three) into one single clonal complex. Interestingly, as reviewed by Feil *et al* [24], nine of the 27 recognized PMEN clones that have contributed to the increase of antimicrobial resistance worldwide (Pneumococcal Molecular Epidemiology Network) [45] are included in this single CC.

In order to discriminate pneumococcal strains within this newly formed CC156, we used a recently developed typing schema based on the sequencing of 96 variable loci belonging to the core genome of *S. pneumoniae* [34]. We found that CC156 can be partitioned into ten genetically and evolutionary distinct lineages homogenous for capsular serotypes and for the presence of PI-1, a genomic region reported to be clonally inherited [26], [27], [43]. Notably, the identified lineages correspond to further partitioning of distinct clonal complexes existing before the identification of ST4945, thus suggesting that these complexes might have originated by artificial grouping due to STs over-sampling. As a demonstration of the higher discriminatory power of 96-MLST, hierarchical clustering, ClonalFrame and phylogenetic analysis of the 96-MLST data resulted in the same grouping of strains, whereas in the case of MLST, probably due to the lower number of probed loci and the different grade of variability of the analysed loci, they did not.

To further support the existence of distinct lineages within CC156, we provide evidence that the diversification of the identified lineages is not due to single recombination events occurring at the level of specific genomic regions, but rather by general sequence variability dispersed along the bacterial chromosome. ST4945 strains were unambiguously assigned to one of the identified lineages (containing also some SLV and DLV of ST4945), suggesting that ST4945 could represent an example of multiple recombination events occurring at the level of MLST loci.

In conclusion, exhaustive MLST typing of large collections of pneumococcal strains has led to the identification of new STs and to the reduction of the discriminatory power of the classical eBURST approach. The analysis of additional loci (such as those included in the 96-MLST schema or of the complete genome) will allow for the reconstruction of the clonal structure and increase the ability to infer evolutionary relationships within the pneumococcal population.

## Supporting Information

Figure S1 Graphic representation of CC156 by e-BURST. A) CC156 is heterogeneous for the presence of PI-1. B) 20 out of the 41 CC156 STs analyzed have three or less than three alleles in common with ST4945. MLST database was accessed on 15h January 2012 and CC156 visualized using eBURST (e-BURST algorithm was run on a dataset comprising all the STs in the database represented once). A) PI-1 presence and PI-1 clade analysis was assessed on all the STs analysed. The STs analysed in this study are highlighted and colour coded based on PI-1 presence as indicated in the Figure. B) The STs analysed in this study are highlighted and colour coded based on the number of 7-MLST alleles in common with ST4945 (colour coding is indicated in the Figure).
(TIF)

Figure S2 96-MLST data analysis (66 strains) by Hierarchical clustering and Clonal Frame. A) Hierarchical clustering performed on the 96-MLST alleles. Numbers are the bootstrap support of each node. B) Consensus network obtained using ClonalFrame on the aligned sequences. The thicker branches have a higher level of statistical support. Lineages are named and highlighted with the same colours of Figure2.
(TIF)

Figure S3 The NJ phylogenetic tree constructed by aligning the 96-MLST concatenated sequences of the 66 CC156 strains analyzed in this study identifies the same 10 lineages (a-j) as the hierarchical clustering (*see* Figure2).
(TIF)

Figure S4 Hierarchical clustering performed on the 7-MLST alleles of the 41 CC156 STs analyzed. Hierarchical clustering was performed using the package Cluster v1.13.1. Distances between strains were computed using the function “Daisy” with Gower’s distance, counting the number of differences between allelic profiles. An agglomerative hierarchical clustering of the data was performed using the function “Agnes” with “average” (unweighted pair-group average method – UPGMA) method.
(TIF)

Figure S5 NJ phylogenetic tree of the 41 CC156 STs analyzed in this study based on the concatenated sequences of the seven MLST loci.
(TIF)

Table S1 Description of the 96-MLST loci set. ID locus name, short description, locus length, coordinates of start and stop on the TIGR4 genome, and number of alleles identified in this study are reported for each locus.
(XLSX)

Table S2 Amplification primers set. For each locus the forward and reverse primers and the PCR amplicon size are indicated.
(XLSX)

Table S3 List of the 96 alleles assigned for each of the 66 strains tested by 96-MLST. Sequences were converted into allelic profiles assigning a progressive unique ID number to each allele. The absent loci were assigned the ID number “0”.
(XLSX)

File S1 Nucleotide sequences of the 96 loci of the 66 strains analyzed in this study.
(TGZ)

## References

[1] O’Brien KL, Wolfson LJ, Watt JP, Henkle E, oria-Knoll M, et al (2009) Burden of disease caused by Streptococcus pneumoniae in children younger than 5 years: global estimates. Lancet 374: 893–902. 19748398 10.1016/S0140-6736(09)61204-6

[2] van der Poll T, Opal SM (2009) Pathogenesis, treatment, and prevention of pneumococcal pneumonia. Lancet 374: 1543–1556. 19880020 10.1016/S0140-6736(09)61114-4

[3] Weiser JN (2010) The pneumococcus: why a commensal misbehaves. J Mol Med 88: 97–102 doi:10.1007/s00109-009-0557-x. 19898768 10.1007/s00109-009-0557-x PMC4487619

[4] Pelton SI, Leibovitz E (2009) Recent advances in otitis media. Pediatr Infect Dis J 28: S133–S137. 19918136 10.1097/INF.0b013e3181b6d81a

[5] Sadowy E, Kuch A, Gniadkowski M, Hryniewicz W (2010) Expansion and evolution of the *Streptococcus pneumoniae* Spain9V-ST156 clonal complex in Poland. Antimicrob Agents Chemother 54: 1720–1727. 20194703 10.1128/AAC.01340-09 PMC2863602

[6] Auranen K, Mehtala J, Tanskanen A, Kaltoft S (2010) Between-strain competition in acquisition and clearance of pneumococcal carriage–epidemiologic evidence from a longitudinal study of day-care children. Am J Epidemiol 171: 169–176. 19969530 10.1093/aje/kwp351 PMC2800239

[7] Lipsitch M, O'Neill K, Cordy D, Bugalter B, Trzcinski K, et al (2007) Strain characteristics of *Streptococcus pneumoniae* carriage and invasive disease isolates during a cluster-randomized clinical trial of the 7-valent pneumococcal conjugate vaccine. J Infect Dis 196: 1221–1227. 17955441 10.1086/521831 PMC3350793

[8] Vainio A, Lyytikainen O, Sihvonen R, Kaijalainen T, Teirila L, et al (2009) An outbreak of pneumonia associated with *S. pneumoniae* at a military training facility in Finland in 2006. APMIS 117: 488–491. 19594488 10.1111/j.1600-0463.2009.02463.x

[9] Antonio M, Hakeem I, Awine T, Secka O, Sankareh K, et al (2008) Seasonality and outbreak of a predominant *Streptococcus pneumoniae* serotype 1 clone from The Gambia: expansion of ST217 hypervirulent clonal complex in West Africa. BMC Microbiol 8: 198 1471-2180-8-198 [pii]; doi:10.1186/1471-2180-8-198 19014613 10.1186/1471-2180-8-198 PMC2587476

[10] Gruber WC, Scott DA, Emini EA (2012) Development and clinical evaluation of Prevnar 13, a 13-valent pneumocococcal CRM197 conjugate vaccine. Ann N Y Acad Sci 1263: 15–26 doi:10.1111/j.1749-6632.2012.06673.x. 22830997 10.1111/j.1749-6632.2012.06673.x

[11] Lucero MG, Dulalia VE, Nillos LT, Williams G, Parreno RA, et al.. (2009) Pneumococcal conjugate vaccines for preventing vaccine-type invasive pneumococcal disease and X-ray defined pneumonia in children less than two years of age. Cochrane Database Syst Rev CD004977. 10.1002/14651858.CD004977.pub2 PMC6464899 19821336

[12] Cutts FT, Zaman SM, Enwere G, Jaffar S, Levine OS, et al (2005) Efficacy of nine-valent pneumococcal conjugate vaccine against pneumonia and invasive pneumococcal disease in The Gambia: randomised, double-blind, placebo-controlled trial. Lancet 365: 1139–1146. 15794968 10.1016/S0140-6736(05)71876-6

[13] Bentley SD, Aanensen DM, Mavroidi A, Saunders D, Rabbinowitsch E, et al (2006) Genetic analysis of the capsular biosynthetic locus from all 90 pneumococcal serotypes. PLoS Genet 2: e31. 16532061 10.1371/journal.pgen.0020031 PMC1391919

[14] Calix JJ, Saad JS, Brady AM, Nahm MH (2012) Structural characterization of *Streptococcus pneumoniae* serotype 9A capsule polysaccharide reveals role of glycosyl 6-O-acetyltransferase wcjE in serotype 9V capsule biosynthesis and immunogenicity. J Biol Chem 287: 13996–14003. M112.346924 [pii]; doi:10.1074/jbc.M112.346924 22367197 10.1074/jbc.M112.346924 PMC3340191

[15] van Cuyck H, Pichon B, Leroy P, Granger-Farbos A, Underwood A, et al (2012) Multiple-Locus Variable-Number Tandem-Repeat Analysis of *Streptococcus pneumoniae* And Comparison with Multiple Loci Sequence Typing. BMC Microbiol 12: 241 1471-2180-12-241 [pii]; doi:10.1186/1471-2180-12-241 23088225 10.1186/1471-2180-12-241 PMC3562504

[16] Pandya GA, McEllistrem MC, Venepally P, Holmes MH, Jarrahi B, et al (2011) Monitoring the long-term molecular epidemiology of the pneumococcus and detection of potential ‘vaccine escape’ strains. PLoS One 6: e15950 doi:10.1371/journal.pone.0015950. 21264340 10.1371/journal.pone.0015950 PMC3018475

[17] Enright MC, Spratt BG (1998) A multilocus sequence typing scheme for *Streptococcus pneumoniae:* identification of clones associated with serious invasive disease. Microbiology 144 (Pt 11): 3049–3060. 10.1099/00221287-144-11-3049 9846740

[18] Ma X, Yao KH, Yu SJ, Zhou L, Li QH, et al (2012) Genotype replacement within serotype 23F *Streptococcus pneumoniae* in Beijing, China: characterization of serotype 23F. Epidemiol Infect 1–7. S0950268812002269 [pii]; doi:10.1017/S0950268812002269 10.1017/S0950268812002269 PMC9155283 23068769

[19] Hanage WP, Bishop CJ, Huang SS, Stevenson AE, Pelton SI, et al (2011) Carried pneumococci in Massachusetts children: the contribution of clonal expansion and serotype switching. Pediatr Infect Dis J 30: 302–308 doi:10.1097/INF.0b013e318201a154. 21085049 10.1097/INF.0b013e318201a154 PMC3175614

[20] Sandgren A, Sjostrom K, Olsson-Liljequist B, Christensson B, Samuelsson A, et al (2004) Effect of clonal and serotype-specific properties on the invasive capacity of *Streptococcus pneumoniae* . J Infect Dis 189: 785–796. JID31327 [pii]. doi:10.1086/381686. 14976594 10.1086/381686

[21] Sjostrom K, Blomberg C, Fernebro J, Dagerhamn J, Morfeldt E, et al (2007) Clonal success of piliated penicillin nonsusceptible pneumococci. Proc Natl Acad Sci U S A 104: 12907–12912. 17644611 10.1073/pnas.0705589104 PMC1929012

[22] Esteva C, Selva L, de Sevilla MF, Garcia-Garcia JJ, Pallares R, et al (2011) *Streptococcus pneumoniae* serotype 1 causing invasive disease among children in Barcelona over a 20-year period (1989–2008). Clin Microbiol Infect 17: 1441–1444 doi:10.1111/j.1469-0691.2011.03526.x. 21729192 10.1111/j.1469-0691.2011.03526.x

[23] Francisco AP, Bugalho M, Ramirez M, Carrico JA (2009) Global optimal eBURST analysis of multilocus typing data using a graphic matroid approach. BMC Bioinformatics 10: 152 1471-2105-10-152 [pii]; doi:10.1186/1471-2105-10-152. 19450271 10.1186/1471-2105-10-152 PMC2705362

[24] Feil EJ, Li BC, Aanensen DM, Hanage WP, Spratt BG (2004) eBURST: inferring patterns of evolutionary descent among clusters of related bacterial genotypes from multilocus sequence typing data. J Bacteriol 186: 1518–1530. 14973027 10.1128/JB.186.5.1518-1530.2004 PMC344416

[25] Willems RJ, Hanage WP, Bessen DE, Feil EJ (2011) Population biology of Gram-positive pathogens: high-risk clones for dissemination of antibiotic resistance. FEMS Microbiol Rev 35: 872–900 doi:10.1111/j.1574-6976.2011.00284.x. 21658083 10.1111/j.1574-6976.2011.00284.x PMC3242168

[26] Aguiar SI, Serrano I, Pinto FR, Melo-Cristino J, Ramirez M (2008) The presence of the pilus locus is a clonal property among pneumococcal invasive isolates. BMC Microbiol 8: 41. 18307767 10.1186/1471-2180-8-41 PMC2270847

[27] Moschioni M, Donati C, Muzzi A, Masignani V, Censini S, et al (2008) *Streptococcus pneumoniae* contains 3 *rlrA* pilus variants that are clonally related. J Infect Dis 197: 888–896. 18269316 10.1086/528375

[28] Moschioni M, De Angelis G, Melchiorre S, Masignani V, Leibovitz E, et al. (2009) Prevalence of pilus encoding islets among acute otitis media *Streptococcus pneumoniae* isolates from Israel. Clin Microbiol Infect. 10.1111/j.1469-0691.2009.03105.x 19886901

[29] Bagnoli F, Moschioni M, Donati C, Dimitrovska V, Ferlenghi I, et al (2008) A second pilus type in *Streptococcus pneumoniae* is prevalent in emerging serotypes and mediates adhesion to host cells. J Bacteriol 190: 5480–5492. 18515415 10.1128/JB.00384-08 PMC2493256

[30] Selva L, Ciruela P, Blanchette K, del Amo E, Pallares R, et al. (2012) Prevalence and clonal distribution of *pcpA*, *psrP* and Pilus-1 among pediatric isolates of *Streptococcus pneumoniae*. PLoS One 7: e41587.;PONE-D-11–08927 [pii]. doi:10.1371/journal.pone.0041587 [doi]. 10.1371/journal.pone.0041587 PMC3404996 22848535

[31] Munoz-Almagro C, Selva L, Sanchez CJ, Esteva C, de Sevilla MF, et al.. (2010) PsrP, a protective pneumococcal antigen, is highly prevalent in children with pneumonia and is strongly associated with clonal type. Clin Vaccine Immunol 17: 1672–1678. CVI.00271–10 [pii]; doi:10.1128/CVI.00271-10. 10.1128/CVI.00271-10 PMC2976083 20861332

[32] Croucher NJ, Harris SR, Fraser C, Quail MA, Burton J, et al.. (2011) Rapid pneumococcal evolution in response to clinical interventions. Science 331: 430–434. 331/6016/430 [pii]; doi:10.1126/science.1198545. 10.1126/science.1198545 PMC3648787 21273480

[33] Donati C, Hiller NL, Tettelin H, Muzzi A, Croucher NJ, et al. (2010) Structure and dynamics of the pan-genome of *Streptococcus pneumoniae* and closely related species. Genome Biol 11: R107. gb-2010-11-10-r107 [pii]; doi:10.1186/gb-2010-11-10-r107. 10.1186/gb-2010-11-10-r107 PMC3218663 21034474

[34] Crisafulli G, Guidotti S, Muzzi A, Torricelli G, Moschioni M, et al. (2012) An extended multi-locus molecular typing schema for *Streptococcus pneumoniae* demonstrates that a limited number of capsular switch events is responsible for serotype heterogeneity of closely related strains from different countries. Infect Genet Evol. S1567-1348(12)00301-2 [pii]; doi:10.1016/j.meegid.2012.09.008. 10.1016/j.meegid.2012.09.008 23022733

[35] Elberse KE, Nunes S, Sa-Leao R, van der Heide HG, Schouls LM (2011) Multiple-locus variable number tandem repeat analysis for *Streptococcus pneumoniae*: comparison with PFGE and MLST. PLoS One 6: e19668. PONE-D-11-00693 [pii]. doi:10.1371/journal.pone.0019668. 10.1371/journal.pone.0019668 PMC3102655 21637335

[36] Rakov AV, Ubukata K, Robinson DA (2011) Population structure of hyperinvasive serotype 12F, clonal complex 218 *Streptococcus pneumoniae* revealed by multilocus boxB sequence typing. Infect Genet Evol 11: 1929–1939. S1567-1348(11)00295-4 [pii]; doi:10.1016/j.meegid.2011.08.016. 10.1016/j.meegid.2011.08.016 PMC3230773 21888992

[37] Edgar RC (2004) MUSCLE: multiple sequence alignment with high accuracy and high throughput. Nucleic Acids Res 32: 1792–1797.;32/5/1792 [pii]. doi:10.1093/nar/gkh340. 10.1093/nar/gkh340 PMC390337 15034147

[38] Kaufman L., Rousseeuw P (2005) [Finding Groups in Data: An Introduction to Cluster Analysis (Wiley Series in Probability and Statistics)]. Wiley-Interscience.

[39] Francisco AP, Vaz C, Monteiro PT, Melo-Cristino J, Ramirez M, et al.. (2012) PHYLOViZ: phylogenetic inference and data visualization for sequence based typing methods. BMC Bioinformatics 13: 87. 1471-2105-13-87 [pii]; doi:10.1186/1471-2105-13-87. 10.1186/1471-2105-13-87 PMC3403920 22568821

[40] Tamura K, Peterson D, Peterson N, Stecher G, Nei M, et al.. (2011) MEGA5: molecular evolutionary genetics analysis using maximum likelihood, evolutionary distance, and maximum parsimony methods. Mol Biol Evol 28: 2731–2739. msr121 [pii]; doi:10.1093/molbev/msr121. 10.1093/molbev/msr121 PMC3203626 21546353

[41] Saitou N, Nei M (1987) The neighbor-joining method: a new method for reconstructing phylogenetic trees. Mol Biol Evol 4: 406–425. 3447015 10.1093/oxfordjournals.molbev.a040454

[42] Didelot X, Falush D (2007) Inference of bacterial microevolution using multilocus sequence data. Genetics 175: 1251–1266. genetics.106.063305 [pii]; doi:10.1534/genetics.106.063305. 10.1534/genetics.106.063305 PMC1840087 17151252

[43] Basset A, Trzcinski K, Hermos C, O'Brien KL, Reid R, et al (2007) Association of the pneumococcal pilus with certain capsular serotypes but not with increased virulence. J Clin Microbiol 45: 1684–1689. 17392439 10.1128/JCM.00265-07 PMC1933072

[44] Siira L, Rantala M, Jalava J, Hakanen AJ, Huovinen P, et al (2009) Temporal trends of antimicrobial resistance and clonality of invasive *Streptococcus pneumoniae* isolates in Finland, 2002 to 2006. Antimicrob Agents Chemother 53: 2066–2073. 19273677 10.1128/AAC.01464-08 PMC2681517

[45] McGee L, McDougal L, Zhou J, Spratt BG, Tenover FC, et al (2001) Nomenclature of major antimicrobial-resistant clones of *Streptococcus pneumoniae* defined by the pneumococcal molecular epidemiology network. J Clin Microbiol 39: 2565–2571 doi:10.1128/JCM.39.7.2565-2571.2001. 11427569 10.1128/JCM.39.7.2565-2571.2001 PMC88185

