# Supplementary figures and images for: Sequence Analysis of 96 Genomic Regions Identifies Distinct Evolutionary Lineages within CC156, the Largest Streptococcus pneumoniae Clonal Complex in the MLST Database
Source: PLoS One. 2013 Apr 12;8(4):e61003. doi: 10.1371/journal.pone.0061003 (PMC3625235; doi:10.1371/journal.pone.0061003)

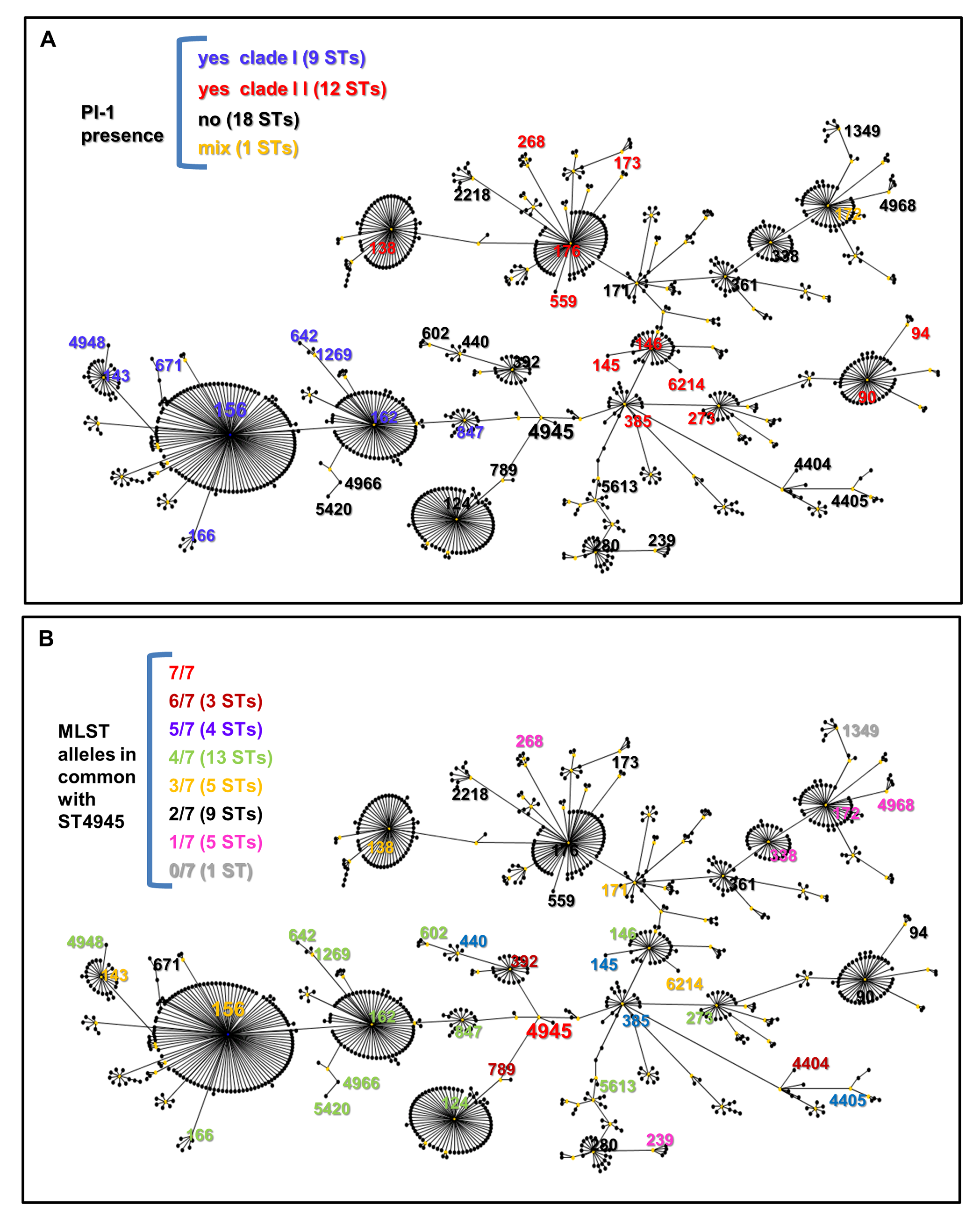

Supplement: Figure S1 — Graphic representation of CC156 by e-BURST. A) CC156 is heterogeneous for the presence of PI-1. B) 20 out of the 41 CC156 STs analyzed have three or less than three alleles in common with ST4945. MLST database was accessed on 15h January 2012 and CC156 visualized using eBURST (e-BURST algorithm was run on a dataset comprising all the STs in the database represented once). A) PI-1 presence and PI-1 clade analysis was assessed on all the STs analysed. The STs analysed in this study are highlighted and colour coded based on PI-1 presence as indicated in the Figure. B) The STs analysed in this study are highlighted and colour coded based on the number of 7-MLST alleles in common with ST4945 (colour coding is indicated in the Figure). (TIF) [file pone.0061003.s001.tif]

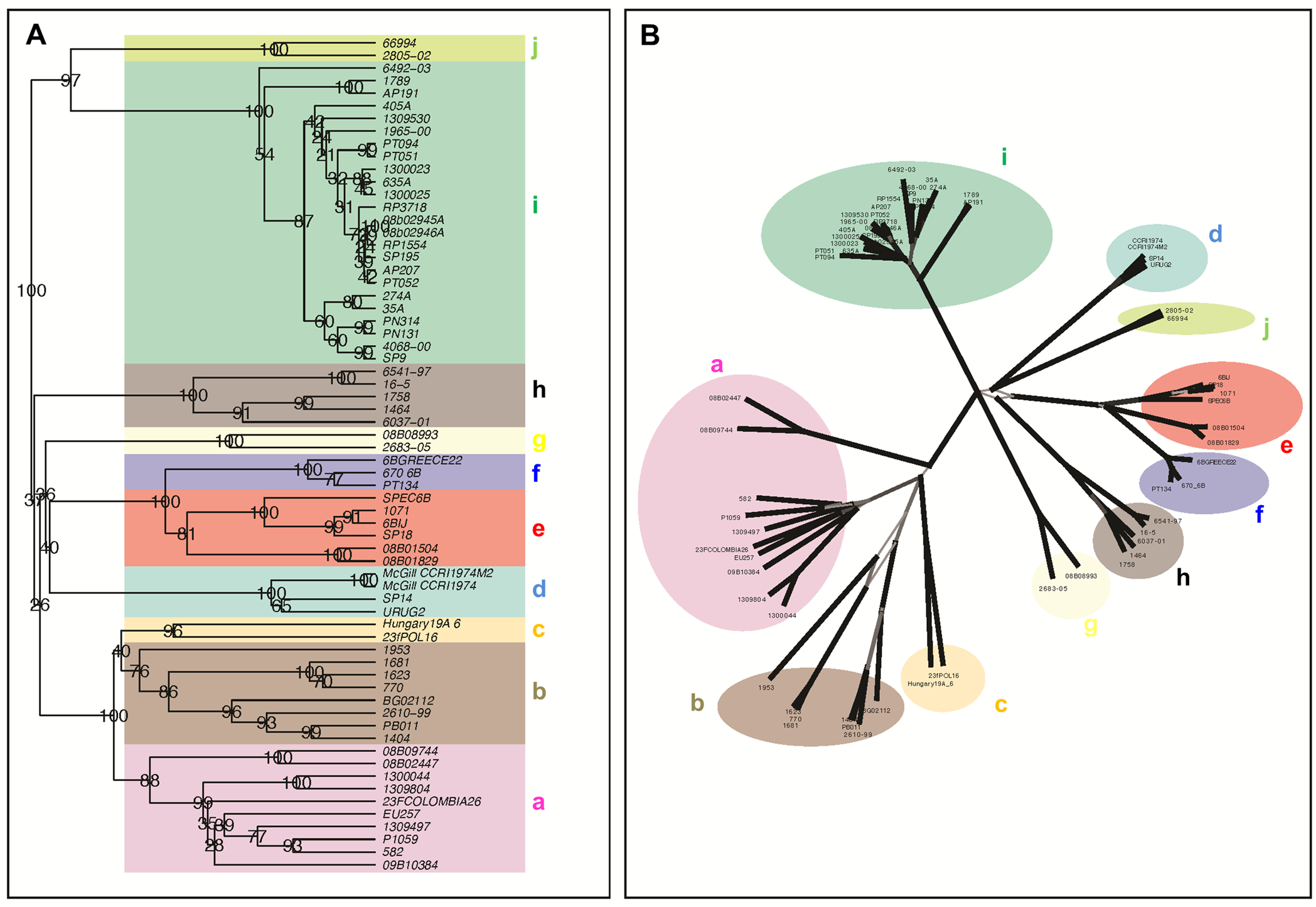

Supplement: Figure S2 — 96-MLST data analysis (66 strains) by Hierarchical clustering and Clonal Frame. A) Hierarchical clustering performed on the 96-MLST alleles. Numbers are the bootstrap support of each node. B) Consensus network obtained using ClonalFrame on the aligned sequences. The thicker branches have a higher level of statistical support. Lineages are named and highlighted with the same colours of Figure2. (TIF) [file pone.0061003.s002.tif]

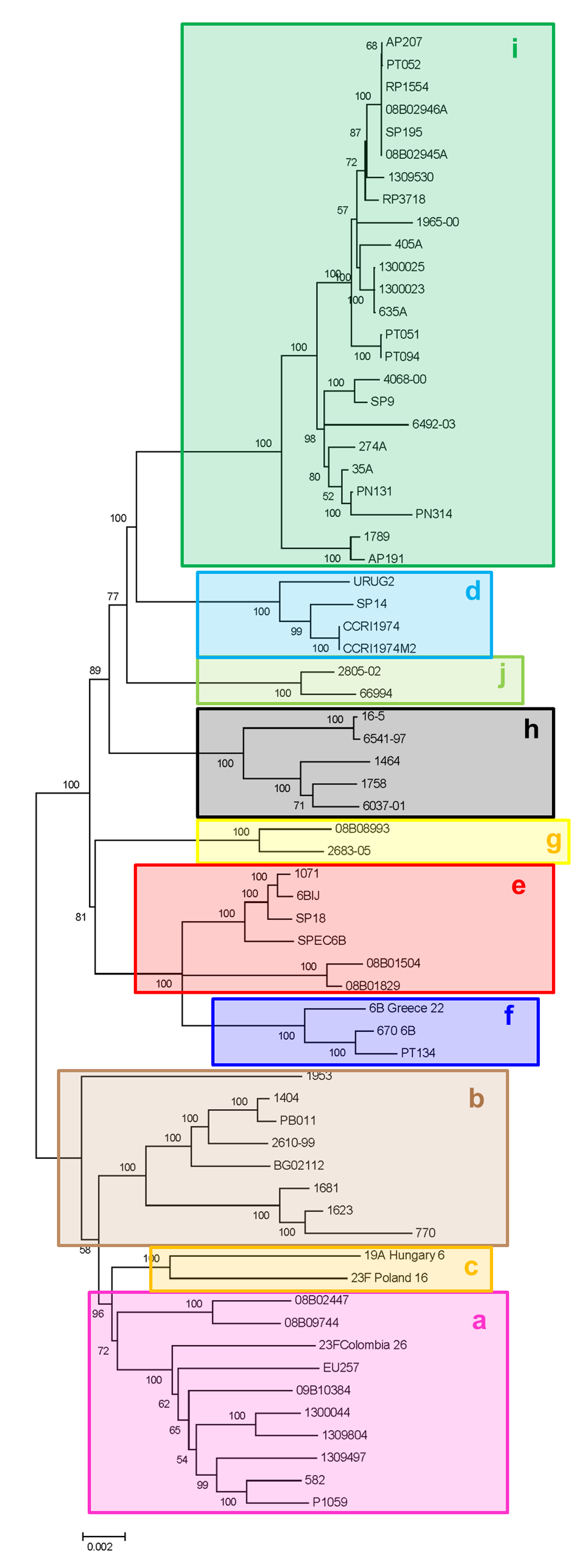

Supplement: Figure S3 — The NJ phylogenetic tree constructed by aligning the 96-MLST concatenated sequences of the 66 CC156 strains analyzed in this study identifies the same 10 lineages (a-j) as the hierarchical clustering (see Figure2). (TIF) [file pone.0061003.s003.tif]

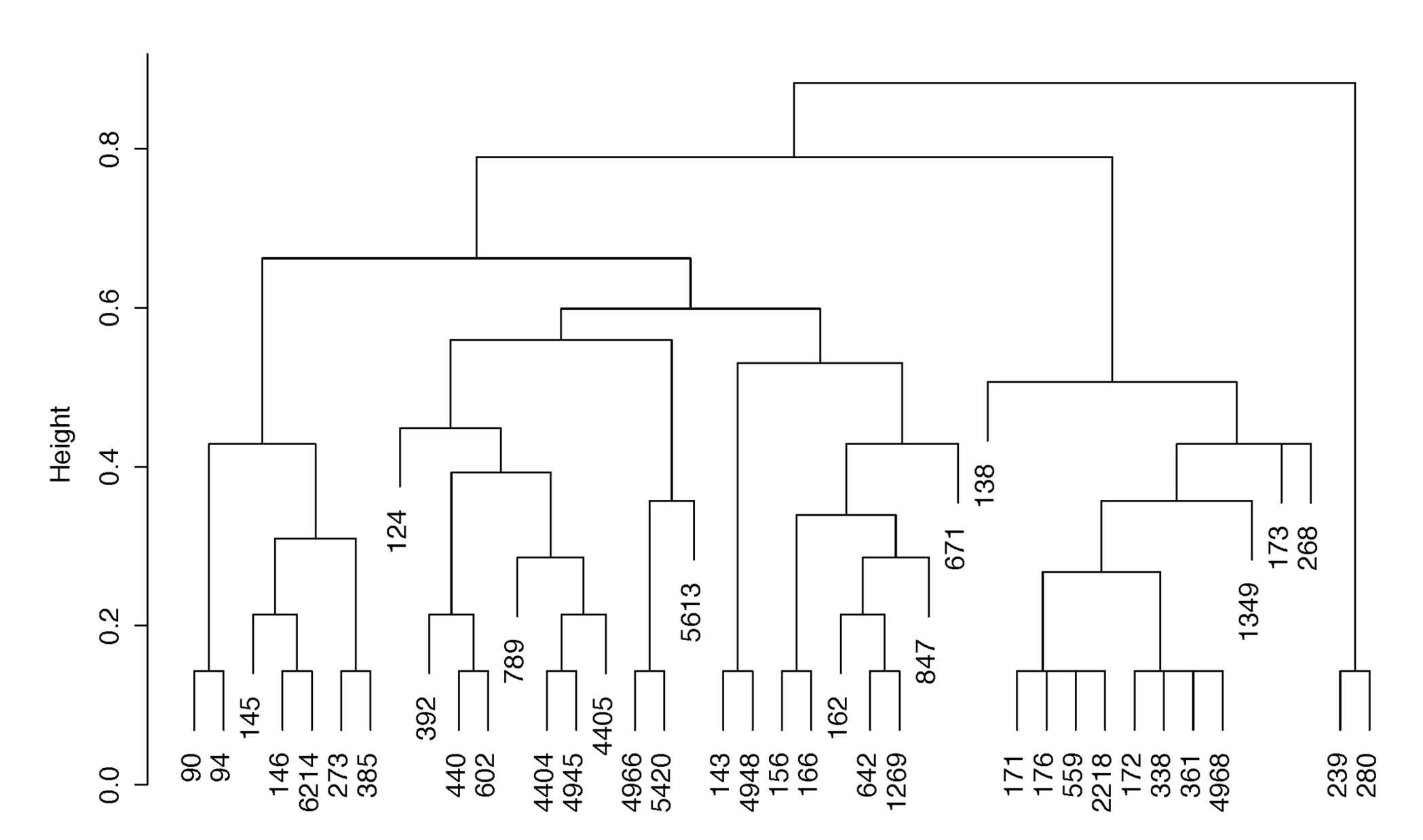

Supplement: Figure S4 — Hierarchical clustering performed on the 7-MLST alleles of the 41 CC156 STs analyzed. Hierarchical clustering was performed using the package Cluster v1.13.1. Distances between strains were computed using the function “Daisy” with Gower’s distance, counting the number of differences between allelic profiles. An agglomerative hierarchical clustering of the data was performed using the function “Agnes” with “average” (unweighted pair-group average method – UPGMA) method. (TIF) [file pone.0061003.s004.tif]
